# Outcomes of COVID-19 in Inflammatory Rheumatic Diseases: A Retrospective Cohort Study

**DOI:** 10.7759/cureus.26343

**Published:** 2022-06-26

**Authors:** Thamer Saad Alhowaish, Moustafa S Alhamadh, Abdulrahman Yousef Alhabeeb, Shaya Fahad Aldosari, Emad Masuadi, Abdulrahman Alrashid

**Affiliations:** 1 Neurology, College of Medicine, King Saud Bin Abdulaziz University for Health Sciences, King Abdullah International Medical Research Center, Ministry of National Guard-Health Affairs, Riyadh, SAU; 2 Internal Medicine, College of Medicine, King Saud Bin Abdulaziz University for Health Sciences, King Abdullah International Medical Research Center, Ministry of National Guard-Health Affairs, Riyadh, SAU; 3 Medicine, King Abdulaziz Medical City, King Abdullah International Medical Research Center, Ministry of National Guard-Health Affairs, Riyadh, SAU; 4 Cardiothoracic Surgery, College of Medicine, King Saud Bin Abdulaziz University for Health Sciences, King Abdullah International Medical Research Center, Ministry of National Guard-Health Affairs, Riyadh, SAU; 5 Research/Biostatistics, King Saud Bin Abdulaziz University for Health Sciences, King Abdullah International Medical Research Center, Riyadh, SAU; 6 Rheumatology, King Abdullah International Medical Research Center, Ministry of National Guard-Health Affairs, Riyadh, SAU

**Keywords:** rheumatic inflammatory disease, rheumatology, inflammatory diseases, immunosuppressants, covid-19

## Abstract

Background

Similar to coronavirus disease 2019 (COVID-19), the pathogenesis of inflammatory rheumatic diseases includes cytokines dysregulation and increased expression of pro-inflammatory cytokines. Although current data from international studies suggest that rheumatic diseases are associated with a higher risk of COVID-19 infection and worse outcomes, there is limited literature in Saudi Arabia. This study aims to evaluate the outcomes and length of hospital stay of COVID-19 patients with inflammatory rheumatic diseases in Saudi Arabia.

Method

This was a single-center retrospective cohort study that included 122 patients with inflammatory rheumatic diseases and documented coronavirus disease 2019 (COVID-19) infection from 2019 to 2021. Patients with suspected COVID-19 infection, non-inflammatory diseases, such as osteoarthritis, or inflammatory diseases but without or with weak systemic involvement, such as gout, were excluded.

Results

The vast majority (81.1%) of the patients were females. Rheumatoid arthritis was the most common primary rheumatological diagnosis. The admission rate was 34.5% with an overall mortality rate of 11.5%. Number of episodes of COVID-19 infection, mechanical ventilation, cytokine storm syndrome, secondary bacterial infection, number of comorbidities, rituximab, diabetes mellitus, hypertension, chronic kidney disease, and heart failure were significantly associated with a longer hospital stay. Additionally, hypertension, heart failure, rituximab, mechanical ventilation, cytokine storm syndrome, and secondary bacterial infection were significantly associated with higher mortality. Predictors of longer hospitalization were obesity, number of episodes of COVID-19 infection, mechanical ventilation, number of comorbidities, and chronic kidney disease, whereas, hypertension was the only predictor of mortality.

Conclusion

Obesity, number of episodes of COVID-19 infection, mechanical ventilation, number of comorbidities, and chronic kidney disease were significantly associated with higher odds of longer hospitalization, whereas, hypertension was significantly associated with higher odds of mortality. We recommend that these patients should be prioritized for the COVID-19 vaccine booster doses, and rituximab should be avoided unless its benefit clearly outweighs its risk.

## Introduction

Since the outbreak of coronavirus disease 2019 (COVID-19), in Wuhan, China, many studies have been conducted to investigate the effect of COVID-19 on the course of multiple diseases. Although it is primarily a respiratory disease that manifests as pneumonia, it could potentially affect other organs and systems including the heart, kidney, gastrointestinal tract, nervous and immune systems, and blood [1].

COVID-19 usually manifests as mild-to-moderate self-limiting respiratory symptoms, such as fever, cough, shortness of breath, and loss of taste and smell. On the other hand, in a severe form of the disease, some patients may require hospitalization and intubation with mechanical ventilation [2,3]. Several factors have been associated with poor outcomes in COVID-19, including old age and preexisting comorbidities, such as diabetes mellitus (DM), hypertension (HTN), and chronic pulmonary diseases [4,5]. Current data suggest that rheumatic diseases impose an additional risk of COVID-19 infection and are associated with poorer outcomes. This risk varies based on the underlying rheumatic disease, comorbidities, and treatments [6].

Autoimmune connective tissue diseases are chronic diseases with female predominance. The most common connective tissue diseases are systemic lupus erythematosus (SLE), scleroderma, myositis, rheumatoid arthritis (RA), and Sjogren’s syndrome [7,8]. The pathogenesis of these conditions is highly complicated, and it includes excessive production of pro-inflammatory cytokines, and therefore, high disease activity could result in flares with severe systemic symptoms and increased inflammatory markers. Similarly, COVID-19 has been associated with cytokine dysregulation and increased expression of pro-inflammatory cytokines, which can cause cytokine storm syndrome (CSS) [9,10]. Furthermore, patients who are already on immunosuppressants are more vulnerable to infection [11,12].

Due to the variability of the results among different studies concerning the outcomes of rheumatic patients with COVID-19, and due to limited literature in Saudi Arabia, we aimed to study the impact of autoimmune connective tissue diseases and immunosuppressants on COVID-19 severity, hospitalization, intensive care unit admission rates, and mortality in Saudi Arabia.

## Materials and methods

Objectives

We sought to evaluate the outcomes (as mortality/survival) and length of hospital stay (if hospitalization was needed) of polymerase chain reaction (PCR)-positive severe acute respiratory syndrome coronavirus 2 (SARS-CoV-2) patients with known inflammatory rheumatic diseases.

Study design/setting

This was a single-center retrospective cohort study that took place in King Abdulaziz Medical City (KAMC), Ministry of National Guard-Health Affairs (MNG-HA), Riyadh, Kingdom of Saudi Arabia. KAMC is an academic government-funded tertiary hospital that combines clinical care, training, academics with research, and state-of-the-art medical technologies.

Inclusion and exclusion criteria

All adult patients with systemic inflammatory rheumatic diseases and PCR-proven COVID-19 infection, from 2019 to 2021 were included. Initially, 192 patients were identified, but after applying the inclusion and exclusion criteria, only 122 were eligible. Patients with suspected COVID-19 infection, non-inflammatory diseases, such as osteoarthritis and fibromyalgia, or inflammatory diseases but without or with weak systemic involvement, such as gout, were excluded.

Data collection

The required data were obtained by screening electronic medical records (via the KAMC electronic system - BestCare; Seoul, South Korea: ezCaretech Co.) of all rheumatology patients who were seen in the clinic or admitted to the hospital from 2019 to 2021. The following data were collected: demographics, comorbidities (such as diabetes mellitus, hypertension, and chronic kidney disease), primary rheumatological diagnosis, symptoms of COVID-19, number of episodes of COVID-19 infection (patients with more than one COVID-19 infection after recovery of the first COVID-19), steroid dose, immunosuppressants, length of admission (in weeks), length of ICU admission, mechanical ventilation, cytokine storm syndrome, secondary bacterial infection, and outcomes (as mortality or survival). To know the number of episodes of COVID-19 infection, reinfection was defined as having a positive PCR test for SARS-CoV-2 after having two negative PCR tests in a previously infected patient. Cytokine storm syndrome was defined as a serum ferritin level of at least 10 µg/L, and secondary bacterial infection was defined as having a positive, respiratory or blood, bacterial culture after COVID-19 diagnosis.

Statistical analysis

Statistical Package for the Social Sciences (SPSS) version 22 (Armonk, NY: IBM Corp.) was used for data analysis. Categorical variables were presented as frequencies and percentages, whereas, numerical variables were presented as mean±standard deviation. Due to the small sample size, Fisher's exact test was used instead of chi-square to test the association between categorical variables, and independent sample t-test was used to test the association between numerical variables. Multivariate logistic regression analysis was done to assess the predictors of COVID-19 infection mortality and hospitalization by calculating the adjusted odds ratios, and odds ratios were reported with 95% confidence interval. A test was considered significant if two-sided p-value was <0.05.

Ethical considerations

The study was approved by the Institutional Review Board of King Abdullah International Medical Research Center, Ministry of National Guard-Health Affairs, Riyadh, Kingdom of Saudi Arabia (#RC20/665/R). Informed consent was waived because of the retrospective nature of this study. Access to the data was restricted to the researchers. The confidentiality of all patients was protected, and no names or medical record numbers were used. Privacy and confidentiality were assured and all the data, both hard and soft copies, were kept in a secure place within the National Guard-Health Affairs premises.

## Results

Demographics

**Figure 1:**
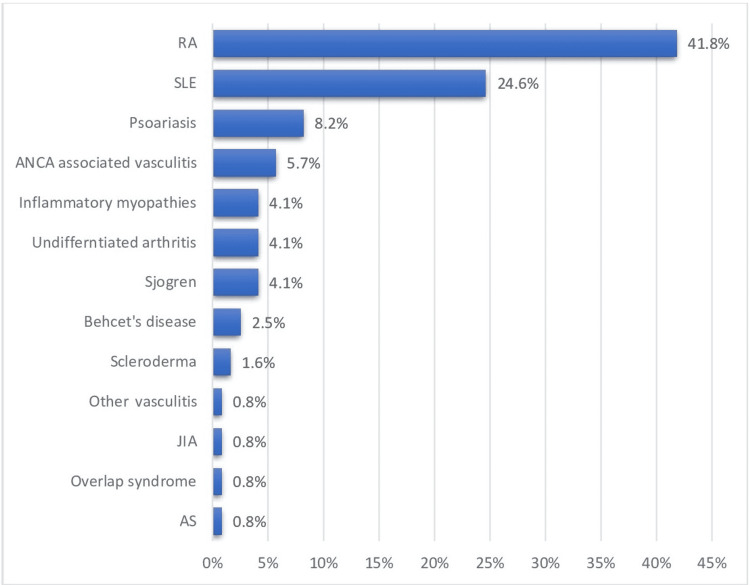
Primary rheumatological diagnoses. RA: rheumatoid arthritis; SLE: systemic lupus erythematosus; JIA: juvenile idiopathic arthritis; AS: ankylosing spondylitis

**Figure 2:**
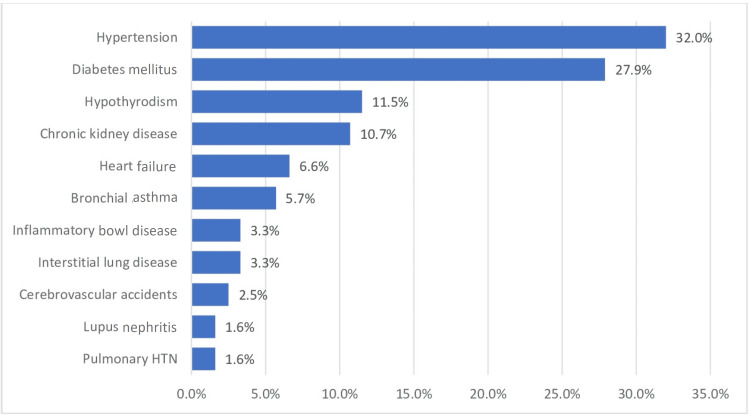
The percentage of each associated comorbidity. The most common comorbidities were hypertension, diabetes mellitus, hypothyroidism, and chronic kidney disease. HTN: hypertension

The demographics of the patients are shown in Table 1. There were a total of 192 rheumatology patients with COVID-19, only 122 of whom were eligible for inclusion. The vast majority (n=99, 81.1%) of the patients were females with a mean age of 48.3±16 years and an average BMI of 30.8±6.4 kg/m^2^. RA, SLE, psoriasis, and antineutrophil cytoplasmic antibodies (ANCA)-positive vasculitis were the most common primary rheumatological diagnoses, accounting for 41.8%, 24.6%, 8.2%, and 5.7% cases, respectively (Figure 1). The most notable associated comorbidities were HTN, DM, hypothyroidism, chronic kidney disease (CKD), heart failure (HF), and bronchial asthma, accounting for 32.0%, 27.9%, 11.5%, 10.7%, 6.6%, and 5.7% cases, respectively (Figure 2).

**Table 1:** Patients’ baseline characteristics, demographics, and associated comorbidities. COVID-19: coronavirus disease 2019; TNF: tumor necrosis factor

Demographics/variables/comorbidities		Values	n	%
Important demographics	Gender	Male	23	18.9%
		Female	99	81.1%
	Age (years)	≤30	13	10.7%
		31-40	32	26.2%
		41-50	24	19.7%
		51-60	24	19.7%
		≥61	29	23.8%
	Body mass index	Underweight	3	2.5%
		Normal weight	17	13.9%
		Overweight	38	31.1%
		Obese	64	52.5%
Hospital-related variables	COVID-19 presenting symptoms	Lower respiratory	59	48.40%
		Upper respiratory	55	45.10%
		Gastrointestinal	13	10.70%
		Other	2	1.60%
	Number of episodes of COVID-19 infection	1	116	95.9%
		2	5	4.1%
	Hospital admission	No admission	80	65.6%
		Admitted for ≤1 week	20	16.4%
		Admitted for >1 week to 4 weeks	14	11.5%
		Admitted for >4 weeks	8	6.6%
	Mechanical ventilation	No	107	87.7
		Yes	14	11.48
	Cytokine storm syndrome	No	118	96.72
		Yes	4	3.28
	Secondary bacterial infections	No	114	93.4%
		Yes	8	6.6%
	Outcome	Survived	108	88.5%
		Died	14	11.5%
	Number of comorbidities	No comorbid condition	52	42.6%
		1	36	29.5%
		2	9	7.4%
		3	17	13.9%
		>3	8	6.5%
Most common comorbidities	Diabetes mellitus		34	27.9%
	Hypertension		39	32.0%
	Hypothyroidism		14	11.5%
	Chronic kidney disease		13	10.7%
	Congestive heart failure		8	6.6%
	Bronchial asthma		7	5.7%
Immunosuppressants and steroids	Hydroxychloroquine		57	46.7%
	Steroid		74	60.7%
	Methotrexate		35	28.7%
	Anti-TNF		12	9.8%
	Mycophenolate		11	9.0%
	Azathioprine		11	9.0%
	Rituximab		6	4.9%
	Tocilizumab (SQ)		6	4.9%
	Tofacitinib		3	2.5%
	Secukinumab		2	1.6%
	Sulfasalazine		2	1.6%
	Abatacept		1	0.8%

Lower respiratory tract symptoms, such as cough and shortness of breath, were the most prominent COVID-19 symptoms with a percentage of 48.4%. Other common COVID-19 presenting symptoms were upper respiratory tract (45.1%) and gastrointestinal symptoms (10.7%). Only five (4.1%) patients had a history of two COVID-19 infections. The majority (65.6%) of the patients did not require hospitalization. However, 16.4% required admission for ≤7 days, 11.5% for eight to 30 days, and 6.6% for >30 days.

The overall mortality rate was 11.5%. A small fraction of the patients (n=17) required ICU admission. Of those, 14 required intubation with mechanical ventilation with a mortality rate of 85.7%. Secondary bacterial infection was only identified in eight (6.6%) patients, four of whom have died. None of the patients who developed CSS (n=4) have survived.

On Fisher's exact test, having more than one COVID-19 infection, intubation with mechanical ventilation, CSS, secondary bacterial infection, and having more than one comorbidity were significantly associated with longer hospital stay (p=0.006, <0.001, 0.006, 0.01, and <0.001, respectively) (Table 2). Moreover, patients with DM, HTN, CKD, and HF were significantly more likely to have longer hospital stay (p=0.001, 0.003, 0.003, and 0.011, respectively). However, only HTN and HF were significantly associated with higher mortality (p=0.002 and 0.006, respectively) (Table 3).

**Table 2:** Inflammatory diseases' effect on hospitalization in association with important baseline characteristics, demographics, associated comorbidities, and immunosuppressants. *P-values were generated by Fisher’s exact test. COVID-19: coronavirus disease 2019; TNF: tumor necrosis factor

Hospital length of stay		Not admitted		≤A week		>A week		p-Values*
		n	%	n	%	n	%	
Gender	Male	13	56.5%	5	21.7%	5	21.7%	0.329
	Female	71	71.7%	13	13.1%	15	15.2%	
Age (years)	≤30	10	76.9%	2	15.4%	1	7.7%	0.406
	31-40	25	78.1%	3	9.4%	4	12.5%	
	41-50	17	70.8%	5	20.8%	2	8.3%	
	51-60	17	70.8%	3	12.5%	4	16.7%	
	≥61	15	51.7%	5	17.2%	9	31.0%	
Body mass index	Underweight	2	66.7%	1	33.3%	0	0.0%	0.857
	Normal weight	13	76.5%	2	11.8%	2	11.8%	
	Overweight	27	71.1%	6	15.8%	5	13.2%	
	Obese	42	65.6%	9	14.1%	13	20.3%	
Steroids	No	32	66.7%	7	14.6%	9	18.8%	0.851
	Yes	52	70.3%	11	14.9%	11	14.9%	
Number of episodes of COVID-19 infection	1	83	71.6%	18	15.5%	15	12.9%	0.006
	2	1	20.0%	0	0.0%	4	80.0%	
Hydroxychloroquine	No	43	66.2%	10	15.4%	12	18.5%	0.825
	Yes	41	71.9%	8	14.0%	8	14.0%	
Mechanical ventilation	No	81	75.7%	15	14.0%	11	10.3%	<0.001
	Yes	3	21.4%	3	21.4%	8	57.1%	
Cytokine storm syndrome	No	84	71.2%	17	14.4%	17	14.4%	0.006
	Yes	0	0.0%	1	25.0%	3	75.0%	
Secondary bacterial infection	No	82	71.9%	16	14.0%	16	14.0%	0.01
	Yes	2	25.0%	2	25.0%	4	50.0%	
Number of comorbidities	0	44	84.6%	5	9.60%	3	5.8%	<0.001
	1	27	75.0%	5	13.90%	4	11.1%	
	2+	13	38.2%	8	23.50%	13	38.2%	
Steroid dose (in mg)	Low (≤5)	70	68.6%	14	13.70%	18	17.6%	0.605
	High (>5)	14	70.0%	4	20.00%	2	10.0%	
Diabetes mellitus	No	69	78.4%	11	12.5%	8	9.1%	0.001
	Yes	15	44.1%	7	20.6%	12	35.3%	
Hypertension	No	65	78.3%	10	12.0%	8	9.6%	0.003
	Yes	19	48.7%	8	20.5%	12	30.8%	
Chronic kidney disease	No	79	73.1%	15	13.9%	14	13.0%	0.003
	Yes	4	30.8%	3	23.1%	6	46.2%	
Hypothyroidism	No	75	69.4%	16	14.8%	17	15.7%	0.908
	Yes	9	64.3%	2	14.3%	3	21.4%	
Interstitial lung disease	No	82	69.5%	18	15.3%	18	15.3%	0.221
	Yes	2	50.0%	0	0.0%	2	50.0%	
Inflammatory bowel disease	No	81	68.6%	18	15.3%	19	16.1%	0.771
	Yes	3	75.0%	0	0.0%	1	25.0%	
Heart failure	No	82	71.9%	16	14.0%	16	14.0%	0.011
	Yes	2	25.0%	2	25.0%	4	50.0%	
Bronchial asthma	No	81	70.4%	15	13.0%	19	16.5%	0.065
	Yes	3	42.9%	3	42.9%	1	14.3%	
Azathioprine	No	75	67.60%	17	15.30%	19	17.10%	0.803
	Yes	9	81.80%	1	9.10%	1	9.10%	
Mycophenolate	No	75	67.60%	17	15.30%	19	17.10%	0.803
	Yes	9	81.80%	1	9.10%	1	9.10%	
Methotrexate	No	59	67.80%	11	12.60%	17	19.50%	0.269
	Yes	25	71.40%	7	20.00%	3	8.60%	
Tocilizumab (SQ)	No	81	69.80%	18	15.50%	17	14.70%	0.119
	Yes	3	50.00%	0	0.00%	3	50.00%	
Rituximab	No	82	70.70%	17	14.70%	17	14.70%	0.046
	Yes	2	33.30%	1	16.70%	3	50.00%	
Anti-TNF	No	77	70.00%	14	12.70%	19	17.30%	0.141
	Yes	7	58.30%	4	33.30%	1	8.30%	

**Table 3:** Inflammatory diseases' outcomes (survival/death) in association with important baseline characteristics, demographics, associated comorbidities, and immunosuppressants. *P-values were generated by Fisher’s exact test. **Based on the Mann-Whitney U test. COVID-19: coronavirus disease 2019; TNF: tumor necrosis factor

Variables		Survival		Mortality		p-Values*
		n	%	n	%	
Gender	Male	17	73.9%	6	26.1%	0.025
	Female	91	91.9%	8	8.1%	
Age (years)	≤30	13	100.0%	0	0.0%	0.07
	31-40	31	96.9%	1	3.1%	
	41-50	22	91.7%	2	8.3%	
	51-60	19	79.2%	5	20.8%	
	≥61	23	79.3%	6	20.7%	
Body mass index	Underweight	3	100.0%	0	0.0%	0.283
	Normal weight	13	76.5%	4	23.5%	
	Overweight	33	86.8%	5	13.2%	
	Obese	59	92.2%	5	7.8%	
Steroids	No	44	91.7%	4	8.3%	0.465
	Yes	64	86.5%	10	13.5%	
Number of episodes of COVID-19 infection	1	103	88.8%	13	11.2%	0.168
	2	4	80.0%	1	20.0%	
Hydroxychloroquine	No	55	84.6%	10	15.4%	0.168
	Yes	53	93.0%	4	7.0%	
Mechanical ventilation	No	105	98.1%	2	1.9%	<0.001
	Yes	2	14.3%	12	85.7%	
Cytokine storm syndrome	No	108	91.5%	10	8.5%	<0.001
	Yes	0	0.0%	4	100.0%	
Secondary bacterial infections	No	104	91.2%	10	8.8%	0.006
	Yes	4	50.0%	4	50.0%	
Hospital length of stay	Not admitted	80	95.2%	4	4.8%	0.001
	Admitted for ≤1 week	15	83.3%	3	16.7%	
	Admitted for >1 week	13	65.0%	7	35.0%	
Number of comorbidities	0	49	94.2%	3	5.8%	0.111
	1	32	88.9%	4	11.1%	
	2+	27	79.4%	7	20.6%	
Steroid dose (in mg)	Low (≤5)	90	88.2%	12	11.8%	0.821/0.831**
	High (>5)	18	90.0%	2	10.0%	
Diabetes mellitus	No	80	90.9%	8	9.1%	0.211
	Yes	28	82.4%	6	17.6%	
Hypertension	No	79	95.2%	4	4.8%	0.002
	Yes	29	74.4%	10	25.6%	
Chronic kidney disease	No	96	88.9%	12	11.1%	0.646
	Yes	11	84.6%	2	15.4%	
Hypothyroidism	No	94	87.0%	14	13.0%	0.366
	Yes	14	100.0%	0	0.0%	
Interstitial lung disease	No	105	89.0%	13	11.0%	0.39
	Yes	3	75.0%	1	25.0%	
Inflammatory bowel disease	No	105	89.0%	13	11.0%	0.39
	Yes	3	75.0%	1	25.0%	
Heart failure	No	104	91.2%	10	8.8%	0.006
	Yes	4	50.0%	4	50.0%	
Bronchial asthma	No	102	88.7%	13	11.3%	0.584
	Yes	6	85.7%	1	14.3%	
Azathioprine	No	98	88.30%	13	11.70%	1
	Yes	10	90.90%	1	9.10%	
Mycophenolate	No	99	89.20%	12	10.80%	0.614
	Yes	9	81.80%	2	18.20%	
Methotrexate	No	76	87.40%	11	12.60%	0.755
	Yes	32	91.40%	3	8.60%	
Sulfasalazine	No	106	88.30%	14	11.70%	
	Yes	2	100.00%	0	0.00%	
Tocilizumab (SQ)	No	103	88.80%	13	11.20%	0.527
	Yes	5	83.30%	1	16.70%	
Rituximab	No	106	91.40%	10	8.60%	0.001
	Yes	2	33.30%	4	66.70%	
Anti-TNF	No	97	88.20%	13	11.80%	1
	Yes	11	91.70%	1	8.30%	

Medications

As a part of their treatment regimen for an underlying rheumatological disease, 60.7% of the patients were on prednisone, 46.7% were on hydroxychloroquine, 28.7% were on methotrexate, 9.8% were on anti-TNF (infliximab or etanercept), 9.0% were on mycophenolate and azathioprine, and 4.9% were on rituximab and tocilizumab. Of the aforementioned immunosuppressants, only rituximab was significantly associated with longer hospitalization and mortality (p=0.046, 0.001). No significance was found between steroid dose and hospital length of stay (p=0.605) or mortality (p=0.821) (Tables 2, 3).

Survival and mortality

Females had more favorable survival compared to males (p=0.025). Intubation with mechanical ventilation, CSS, secondary bacterial infection, and hospital length stay were associated with higher mortality rates (p≤0.001, <0.001, 0.006, and 0.001, respectively). Having a higher number of comorbidities was not associated with higher mortality (p=0.11) (Table 3).

Multivariate regression analysis

In multivariate regression model, obesity (odds ratio {OR}=60.669, 95% confidence interval {CI} 3.53-1042.413, p=0.005), number of COVID-19 infection (OR=59.08, 95% CI 2.532-1378.362, p=0.011), intubation with mechanical ventilation (OR=23.238, 95% CI 3.15-171.434, p=0.002), number of comorbidities (OR=7.11, 95% CI 1.911-26.454, p=0.003), CKD (OR=6.178, 95% CI 1.706-22.38, p=0.006), and HTN (OR=5.291,95% CI 1.266-22.112, p=0.022) were significantly associated with higher odds of hospitalization (Table 4). The only comorbidity that was significantly associated with higher odds of mortality was HTN (OR=5.291, 95% CI 1.266-22.112, p=0.022) (Table 5).

**Table 4:** Ordinal regression model examining the association between important demographics and comorbidities with COVID-19 hospitalization in inflammatory diseases. COVID-19: coronavirus disease 2019

Variables		Odds ratio (95% confidence interval)	p-Values
Gender	Female	0.557 (0.155-2.007)	0.371
	Male	Ref.	Ref.
Age (years)	31-40	0.748 (0.167-3.359)	0.705
	41-50	0.811 (0.273-3.813)	0.791
	51-60	1.15 (0.265-4.997)	0.852
	≥61	Ref.	Ref.
Body mass index	Normal	60.669 (3.532-1042.413)	0.005
	Overweight	1.12 (0.339-3.704)	0.852
	Obese	Ref.	Ref.
Number of episodes of COVID-19 infection	1	59.08 (2.532-1378.362)	0.011
	2	Ref.	Ref.
Steroids	No	0.426 (0.13-1.398)	0.159
	Yes	Ref.	Ref.
Hydroxychloroquine	No	0.994 (0.333-2.97)	0.992
	Yes	Ref.	Ref.
Mechanical ventilation	No	23.238 (3.15-171.434)	0.002
	Yes	Ref.	Ref.
Cytokine storm syndrome	No	6.53 (0.175-244.279)	0.31
	Yes	Ref.	Ref.
Secondary bacterial infection	No	4.251 (0.697-25.913)	0.117
	Yes	Ref	Ref.
Number of comorbidities	0	7.11 (1.911-26.454)	0.003
	1	3.697 (1.003-13.631)	0.05
	+2	Ref.	Ref.
Steroid dose (in mg)	Low (≤5)	3.325 (0.746-14.827)	0.115
	High (>5)	Ref.	Ref.
Diabetes mellitus	Yes	2.565 (0.92-7.149)	0.072
	No	Ref.	Ref.
Hypertension	Yes	2.075 (0.767-5.619)	0.151
	No	Ref.	Ref.
Chronic kidney disease	Yes	6.178 (1.706-22.38)	0.006
	No	Ref.	Ref.
Hypothyroidism	Yes	0.617 (0.149-2.56)	0.506
	No	Ref.	Ref.
Interstitial lung disease	Yes	4.084 (0.406-41.094)	0.232
	No	Ref.	Ref.
Inflammatory bowel disease	Yes	1.195 (0.104-13.671)	0.886
	No	Ref.	Ref.
Heart failure	Yes	1.743 (0.331-9.176)	0.512
	No	Ref.	Ref.
Bronchial asthma	Yes	1.597 (0.324-7.879)	0.565
	No	Ref.	Ref.

**Table 5:** Binary regression model examining the association of important comorbidities with the outcomes (survival/death) of COVID-19 in inflammatory rheumatic diseases. COVID-19: coronavirus disease 2019

Variables	Odds ratio (95% confidence interval)	p-Values
Diabetes mellitus	0.41 (0.074-2.28)	0.309
Hypertension	5.291 (1.266-22.112)	0.022
Chronic kidney disease	1.057 (0.132-8.475)	0.958
Hypothyroidism	0.447 (0.043-4.649)	0.5
Interstitial lung disease	3.642 (0.28-47.386)	0.323
Inflammatory bowel disease	2.009 (0.119-34.016)	0.629
Heart failure	5.933 (0.812-43.347)	0.079
Bronchial asthma	1.082 (0.078-15.096)	0.953

## Discussion

Autoimmune connective tissue diseases are chronic inflammatory diseases with highly complicated pathogenesis that includes excessive production of pro-inflammatory cytokines. Similarly, COVID-19 has been associated with cytokine dysregulation and increased expression of proinflammatory cytokines [9-11]. Patients who are already on immunosuppressive medications are logically more vulnerable to infections [11,12]. Current data suggest that rheumatic diseases are associated with an additional risk of COVID-19 infection and poorer outcomes [6]. In this study, we explored the impact of autoimmune connective tissue diseases and immunosuppressive medications on COVID-19 severity, hospitalization, intensive care unit admission, and mortality rates in Saudi Arabia.

Our patients had a mean age of 48.3±16 years with females being predominant (81.1%). This is attributed to the fact that inflammatory autoimmune diseases generally have female predilection [7,8]. This is in accordance with other studies, as D’Silva et al. who studied the outcomes of 52 COVID-19-infected patients with rheumatic diseases, also reported female predominance. Compared to previously published studies, our patients had a relatively younger mean age [13,14]. Overall hospital mortality of COVID-19 is generally between 15% and 20% and can reach up to 60% in older patients. However, it highly varies across cohorts, reflecting differences in the completeness of testing and case identification, variable thresholds for hospitalization, and differences in outcomes [15-17]. Hospital mortality ranges from less than 5% in patients younger than 40 years to 35% in 70-79 years and greater than 60% in 80-89 years [18]. In our study, the mortality rate was 11.5%, and the mean age was 48.3 which is in compliance with some of the studies. To clarify, Montero et al. reported a mortality rate of 16% [12]. The two percentages are close, and probably our study would have a higher mortality rate if it was delayed further. In contrast, Sharmeen et al. mentioned a mortality rate of 5.9% [19]. Although both Montero and Sharmeen studies have published their works in August 2020, the mortality rates are utterly different. It is hard to judge whether, for example, patients with low mortality rates have been vaccinated and therefore had a milder form of the disease or specific immunosuppressive regimen could have protected those patients. Another factor that could potentially contribute to the differences in mortality rate is the mean age. In our study, the mean age was 48.3 years, whereas, in Montero and Sharmeen they were 60.9 and 57 years, respectively [12,19]. This could not explain the low mortality rate reported in Sharmeen's study. It is also important to mention that our mortality rate might not reflect the actual percentage due to the small sample size and the following limitations: 1) we do not have a unified database for all patients throughout Saudi Arabia and so we could not include patients from other hospitals. 2) Many patients were non-eligible for follow-up in our institution (MNG-HA, KAMC), and so, they might have died outside our institution. 4) Many patients might have died after we collected the data. 3) Many patients, even if eligible, lives outside Riyadh and so cannot be followed up. In our country, Saudi Arabia, at least 56,707,289 doses of COVID vaccines have been administered so far though the mortality rate in our study is still high [20].

The need for admission of COVID-19 patients in the general population depends mainly on their age and preexisting comorbidities, such as chronic respiratory diseases and DM [21,22]. The likelihood of hospitalization increases with age up to a maximum of 18.4% in patients ≥80 years old [23]. In our study, the admission rate was 35%, which is much higher than the global admission rate of the general population. This high percentage could partially be explained by the fact that we included all rheumatology patients with documented COVID-19 from 2019 to 2021. At the beginning of the pandemic, with the lack of clear guidelines, institutions tended to admit COVID-19 positive patients till their swaps came negative. This is a possible explanation for the high admission rate seen in our study. Previously published studies are in agreement with our high admission rate. To emphasize, Gianfrancesco et al. reported an admission rate of 46% [15]. Similarly, Montero et al. also mentioned a high admission rate that is 68% [12]. In addition to what we mentioned above, another explanation could be disease-specific factors as patients with inflammatory diseases might need more medical attention. This is not only limited to rheumatology patients, it is also seen with other autoimmune diseases. To clarify, Sahraian et al. reported a hospitalization rate of 25% in multiple sclerosis patients infected with COVID-19, which is also much higher than the admission rate of the general population in the age group associated with multiple sclerosis patients [24].

In our study, number of COVID infections, CSS, secondary bacterial infection, number of comorbidities, DM, HTN, CKD, and HF were significantly associated with a longer hospital stay. A lot of these factors are in agreement with other studies. For example, D’Silva et al. reported several factors that have been significantly associated with longer hospital stay including older age, number of comorbidities, and DM [14]. Moreover, Stradner et al. also reported the same thing. They found that old age and comorbidities, such as HTN, DM, cardiovascular and pulmonary diseases, and end-stage kidney disease were significantly associated with longer hospitalization [25].

Some reports found that rituximab use is not associated with worse outcomes or course of disease in patients with COVID-19. In our study, the only medication that was significantly associated with longer hospitalization and higher mortality was rituximab. Similarly, Tepasse et al., Stradner et al., and Alpizar-Rodriguez et al., in their studies, concluded that rituximab is associated with a higher risk of severe disease and/or mortality in patients with COVID-19 infection [25-27]. Ideally, immunoglobulin levels should be obtained in all patients prior to rituximab prescription. Unfortunately, to the best of our knowledge, our institution does not mandate immunoglobulin levels prior to rituximab prescription, which could explain the high mortality rate and hospitalization in our study. Though it is crucial to keep in mind that our findings are consistent with the literature [25-27]. Possibly due to the small sample size, we have not found any significance with steroid use nor with other immunosuppressants. However, in Gianfrancesco's study, prednisone ≥10 mg/day was associated with a higher hospitalization rate. Conversely, it has been found that TNF-α inhibitor use was associated with less hospitalization rate [15].

The susceptibility to and severity of COVID-19 is highly influenced by patients’ comorbidities, such as hypertension, and dysregulated innate immune response as in patients with inflammatory autoimmune diseases [9,11,12,28,29]. This might be due to enhanced expression of angiotensin-converting enzyme 2 (ACE2) receptors on the surface of several organs and epithelial cells. COVID-19 infects epithelial cells through binding with ACE2 and initiates inflammation, endothelial activation, tissue damage, and disordered cytokine release [29,30]. Although, in our study, all the included patients were known to have inflammatory rheumatologic diseases, according to literature, those patients are more likely to be infected with and to develop severe COVID-19. To emphasize, D’Silva et al. reported that in COVID-19 patients, the need for intubation with mechanical ventilation was more common in patients with known rheumatologic diseases compared to the general population. Patients with autoimmune inflammatory diseases already have high cytokines and immune dysregulation [14]. The high levels of cytokines intensify the destructive progression that leads to additional epithelial cells dysfunction and inflammation [29,31,32]. Altogether, these disorders ultimately lead to multi-organ failure and death. Comorbidities and suppressed immunity have been found as primary reasons for the exacerbated rate of infection and mortality of COVID-19 [29,30,33]. This is another explanation for the high mortality rate as a lot of those patients are chronically on immunosuppressants. In COVID-19 patients, cellular immunity fails to provide adequate protection due to the virus’s ability to escape the innate immunity and induce a functional decline in T-cell counts [29]. The literature identifies TNF-α and IL-6 receptor inhibitors to be effective in treating COVID-19 among patients with rheumatic diseases as during recovery of COVID-19, decreased levels of IL-6 and TNF-α increase the total T-cell counts [34,35]. In our study, we have not found any protective role for TNF-α and IL-6 receptor inhibitors, probably due to the small sample size.

The studied population should be prioritized for the booster dose of COVID-19 vaccine. Those patients are particularly at increased risk of severe infection, and so they should have more precautions. Rituximab should be avoided unless it is the only option with the benefit clearly outweighing the risk. Prompt seeking medical attention is also recommended to prevent morbidity and mortality.

This study is mainly affected by its single-centered retrospective design and the small sample size. The small sample size limited our statistical analysis as we could not perform Kaplan-Meier survival curve. The results could have been affected by the fact that vaccination-related data were not available and so the effect of vaccination on patients’ outcomes was neglected in the study. We plan to do a follow-up study to assess the effect of vaccination on the outcomes of inflammatory rheumatic diseases.

## Conclusions

Over a third (34.5%) of the patients required hospital admission. Predictors of longer hospitalization were obesity, number of COVID-19 infections, mechanical ventilation, number of comorbidities, HTN, and CKD, whereas, HTN was the only predictor for mortality. Furthermore, rituximab was significantly associated with longer hospitalization and higher mortality. Based on what we found, we recommend that patients with inflammatory rheumatic diseases should be prioritized for the COVID-19 vaccine booster dose, and rituximab should be avoided unless its benefit clearly outweighs its risk.
